# Supplementary figures and images for: Survival and complications after neoadjuvant chemoradiotherapy versus neoadjuvant chemotherapy for esophageal squamous cell cancer: A meta-analysis
Source: PLoS One. 2022 Aug 5;17(8):e0271242. doi: 10.1371/journal.pone.0271242 (PMC9355212; doi:10.1371/journal.pone.0271242)

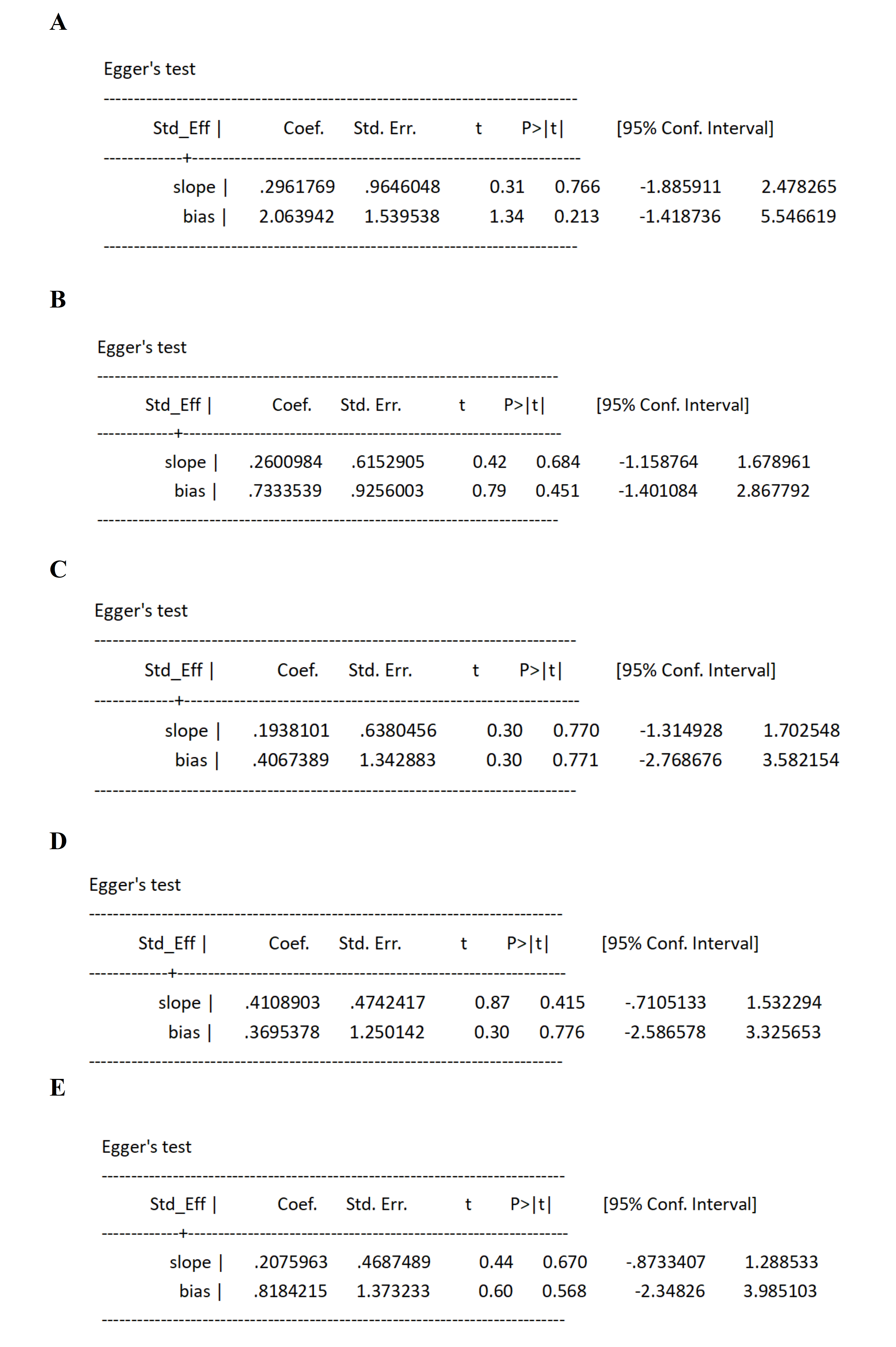

Supplement: S1 Fig — Egger’s tests for publication bias of pCR (A), R0 resection (B), 1-year survival rates (C), 3-year survival rates (D) and 5-year survival rates (E) of NCRT group and NCT group. (TIF) [file pone.0271242.s001.tif]

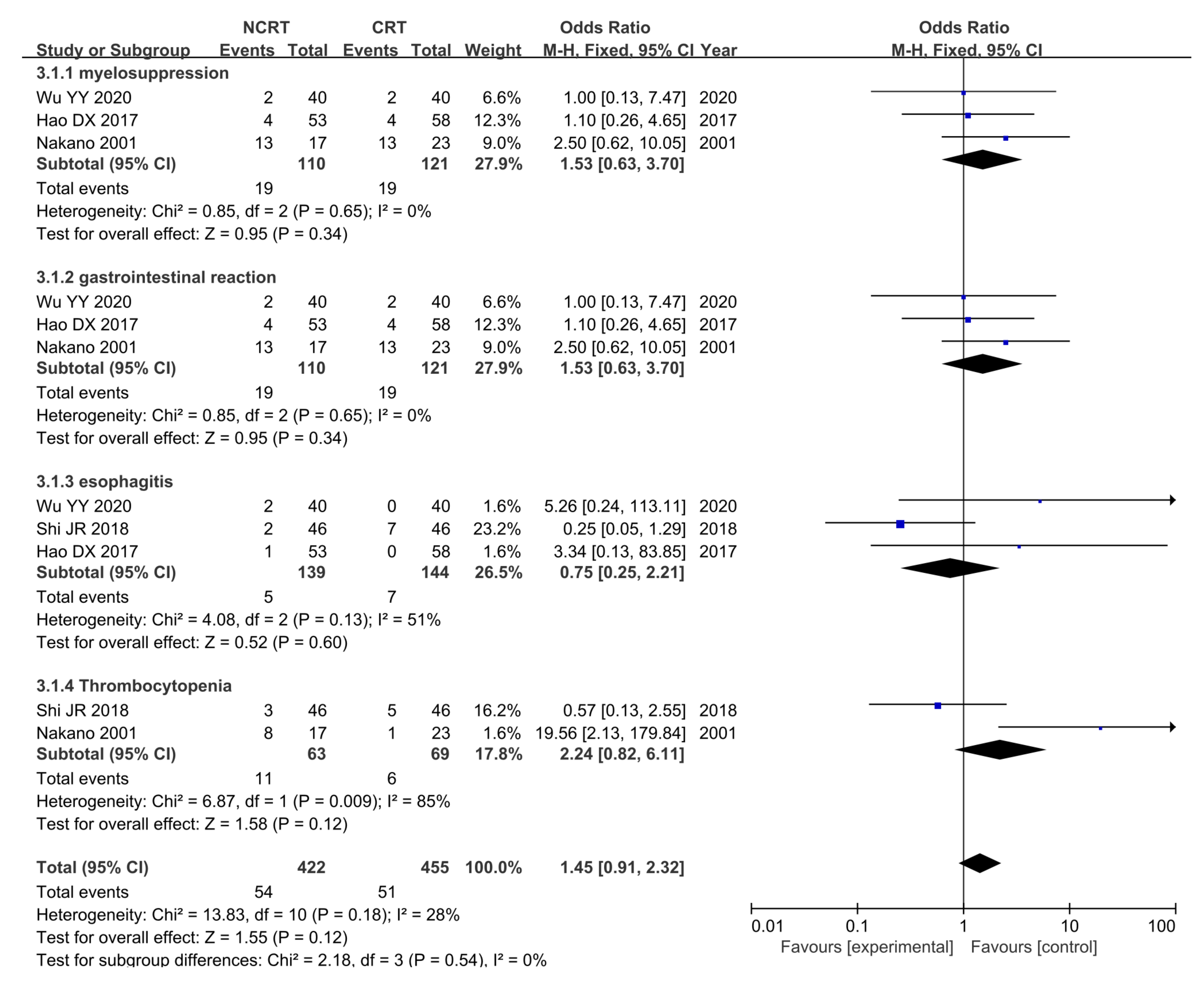

Supplement: S2 Fig — Forest plot for myelosuppression (A), gastrointestinal reaction (B), esophagitis (C) between NCRT group and NCT group. (TIF) [file pone.0271242.s002.tif]

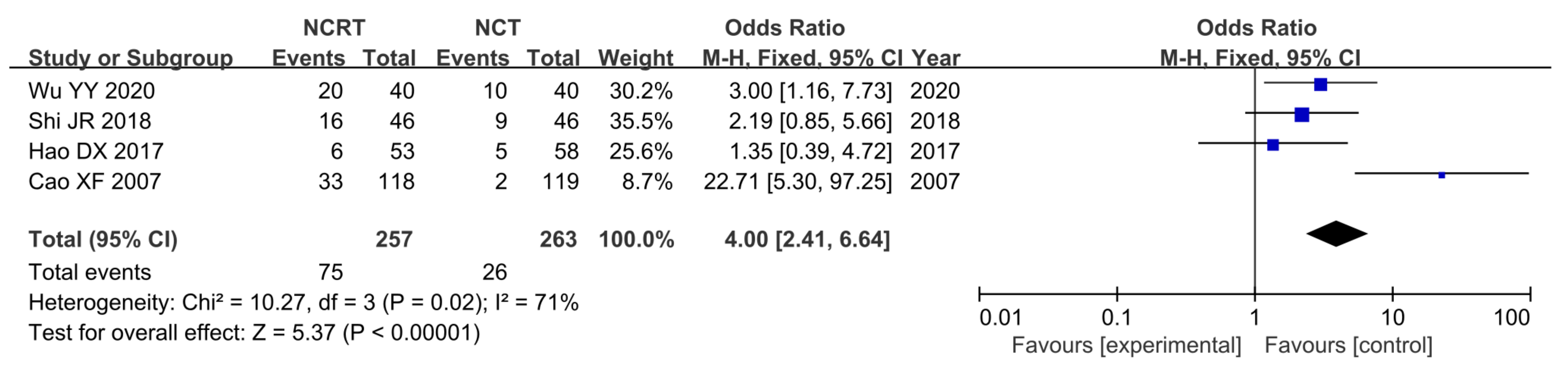

Supplement: S3 Fig — (TIF) [file pone.0271242.s003.tif]
