# Supplementary material for: Survival and complications after neoadjuvant chemoradiotherapy versus neoadjuvant chemotherapy for esophageal squamous cell cancer: A meta-analysis
Source: PLoS One. 2022 Aug 5;17(8):e0271242. doi: 10.1371/journal.pone.0271242 (PMC9355212; doi:10.1371/journal.pone.0271242)
Supplement: S2 Checklist — (DOC) [file pone.0271242.s005.doc]

**PRISMA-P (Preferred Reporting Items for Systematic review and Meta-Analysis Protocols) 2015 checklist: recommended items to address in a systematic review protocol***

| Section and topic | Item No | Checklist item |
| --- | --- | --- |
| ADMINISTRATIVE INFORMATION | | |
| Title: |  | Survival and complications after Neoadjuvant Chemoradiotherapy versus Neoadjuvant Chemotherapy for Esophageal Squamous Cell Cancer：a meta-analysis |
| Identification | 1a | Yes |
| Update | 1b | No |
| Registration | 2 | This protocol was registered with the International Platform of Registered Systematic Review and Meta-Analysis Protocols (INPLASY) on December 05, 2021, and was last updated on December 05, 2021 (registration number INPLASY2021120031). |
| Authors: |  |  |
| Contact | 3a | Yaru Guo,Department of Radiation, the Affiliated Hospital of Xuzhou Medical University,Jiangsu,China. First Clinical College, Xuzhou Medical University, Xuzhou, [China,1768675516@qq.com](mailto:China,1768675516@qq.com)  Mingna Xu,Department of Radiation, the Affiliated Hospital of Xuzhou Medical University,Jiangsu,China. First Clinical College, Xuzhou Medical University, Xuzhou, [China,1198551962@qq.com](mailto:China,1768675516@qq.com)  Yufei Lou,Department of Radiation, the Affiliated Hospital of Xuzhou Medical University,Jiangsu,China. First Clinical College, Xuzhou Medical University, Xuzhou, [China,583936230@qq.com](mailto:China,1768675516@qq.com)  Yan Yuan,Department of Radiation, the Affiliated Hospital of Xuzhou Medical University,Jiangsu,China. First Clinical College, Xuzhou Medical University, Xuzhou, [China,y18752117028@qq.com](mailto:China,1768675516@qq.com)  Longzhen Zhang,Department of Radiation, the Affiliated Hospital of Xuzhou Medical University,Jiangsu, [China,jsxzzlz@126.com](mailto:China,1768675516@qq.com)  Yuling Wu,Department of Radiation, the Affiliated Hospital of Xuzhou Medical University,Jiangsu,China. First Clinical College, Xuzhou Medical University, Xuzhou, [China,1584485401@qq.com](mailto:China,1768675516@qq.com)  Yong Xin,Department of Radiation, the Affiliated Hospital of Xuzhou Medical University,Jiangsu, China. First Clinical College, Xuzhou Medical University, Xuzhou[China,2286840865@qq.com](mailto:China,1768675516@qq.com). Department of Radiotherapy, Xuzhou Medical University, Kunpeng Road, Jiawang District, Xuzhou City, Jiangsu Province, China  Fengjuan Zhou,Department of Radiation,the Second Affiliated Hospital of Xuzhou Medical University, Jiangsu, China First Clinical College, Xuzhou Medical University, Xuzhou, [China,2175170762@qq.com](mailto:China,1768675516@qq.com). General Hospital of Xuzhou Mining Group, No. 32, Meijian Road, Quanshan District, Xuzhou City, Jiangsu Province, China |
| Contributions | 3b | Data curation: Yaru Guo, Mingna Xu, Yufei Lou.  Formal analysis: Yufei Lou, Yan Yuan.  Methodology: Yaru Guo.  Resources: Mingna Xu, Yufei Lou, Yan Yuan.  Writing – original draft: Yaru Guo, Mingna Xu.  Writing – review & editing: Yaru Guo, Mingna Xu, Yufei Lou, Yan Yuan, Yuling Wu.  Fengjuan Zhou is the guarantor of the review |
| Amendments | 4 | No |
| Support: |  |  |
| Sources | 5a | National Natural Science Foundation of China (Grant No. 81972845). |
| Sponsor | 5b | Longzhen Zhang |
| Role of sponsor or funder | 5c | Director Zhang Longzhen provided us with financial support. |
| INTRODUCTION | | |
| Rationale | 6 | Neoadjuvant chemoradiation (NCRT) and neoadjuvant chemotherapy (NCT) have been shown to improve survival in patients with locally advanced esophageal squamous cell carcinoma (ESCC). Neoadjuvant therapy plus surgery has been used as the standard treatment strategy for patients with locally advanced ESCC. The Japanese guidelines favor NCT, while NCCN guidelines recommend NCRT as the first-line treatment option for advanced ESCC .The superiority of either NCRT or NCT in the treatment of esophageal cancer remains controversial.Accumulating evidence shows that Esophageal adenocarcinoma (EAC) and ESCC have different natural histories and cellular origins.We believe that these pathological subtypes should be analyzed separately when attempting to determine the best neoadjuvant treatment for esophageal cancer subtypes. Therefore, we collected all the experiments on NCT and NCRT for ESCC, including randomized controlled trials and retrospective experiments. This study aimed to systematically evaluate the survival and complications following NCRT and NCT for ESCC in meta-analysis and to provide evidence to guide the treatment of ESCC. |
| Objectives | 7 | To compare the survival and complications of neoadjuvant chemoradiation (NCRT) versus neoadjuvant chemotherapy (NCT) for esophageal squamous cell carcinoma (ESCC). |
| METHODS | | |
| Eligibility criteria | 8 | (i) Diagnosis of ESCC following cytological and histopathological examination in patients without serious cardiac, pulmonary, hepatic, or renal disease.  (ii)RCTs or Retrospective experiments comparing NCRT and NCT for treating ESCC.  (iii)The experimental design met the requirements and included patients with ESCC and EAC, and a subgroup analysis was performed with ESCC results reported separately.  (iv)The primary efficacy outcomes were pathological complete remission rate (pCR); complete (R0) tumor resection rate; 1-, 3-, and 5-year survival rates; toxicity of neoadjuvant treatment (including myelosuppression, gastrointestinal reaction, and esophagitis); and postoperative complications (including anastomotic leak, pulmonary complications, cardiac complications, chyle leak, and perioperative mortality). |
| Information sources | 9 | We identified all studies comparing NCRT and NCT in the treatment of EC in the PubMed, Cochrane Library, Web of Science, Embase, Wanfang Data, Chinese National Knowledge Infrastructure (CNKI), Chinese Biological Medicine (CBM) Database, and VIP Database published before November 2021. |
| Search strategy | 10 | (("Esophageal Neoplasms"[Mesh]) OR (((((((((((((((((Esophageal Neoplasm[Title/Abstract]) OR (Neoplasm, Esophageal[Title/Abstract])) OR (Esophagus Neoplasm[Title/Abstract])) OR (Esophagus Neoplasms[Title/Abstract])) OR (Neoplasm, Esophagus[Title/Abstract])) OR (Neoplasms, Esophagus[Title/Abstract])) OR (Neoplasms, Esophageal[Title/Abstract])) OR (Cancer of Esophagus[Title/Abstract])) OR (Cancer of the Esophagus[Title/Abstract])) OR (Esophagus Cancer[Title/Abstract])) OR (Cancer, Esophagus[Title/Abstract])) OR (Cancers, Esophagus[Title/Abstract])) OR (Esophagus Cancers[Title/Abstract])) OR (Esophageal Cancer[Title/Abstract])) OR (Cancer, Esophageal[Title/Abstract])) OR (Cancers, Esophageal[Title/Abstract])) OR (Esophageal Cancers[Title/Abstract]))) AND ((((("Chemoradiotherapy"[Mesh]) OR (((((((((((((((((((((((Chemoradiotherapies[Title/Abstract]) OR (Radiochemotherapy[Title/Abstract])) OR (Radiochemotherapies[Title/Abstract])) OR (Concurrent Chemoradiotherapy[Title/Abstract])) OR (Chemoradiotherapies, Concurrent[Title/Abstract])) OR (Chemoradiotherapy, Concurrent[Title/Abstract])) OR (Concurrent Chemoradiotherapies[Title/Abstract])) OR (Synchronous Chemoradiotherapy[Title/Abstract])) OR (Chemoradiotherapies, Synchronous[Title/Abstract])) OR (Chemoradiotherapy, Synchronous[Title/Abstract])) OR (Synchronous Chemoradiotherapies[Title/Abstract])) OR (Concurrent Radiochemotherapy[Title/Abstract])) OR (Concurrent Radiochemotherapies[Title/Abstract])) OR (Radiochemotherapies, Concurrent[Title/Abstract])) OR (Radiochemotherapy, Concurrent[Title/Abstract])) OR (Concomitant Chemoradiotherapy[Title/Abstract])) OR (Chemoradiotherapies, Concomitant[Title/Abstract])) OR (Chemoradiotherapy, Concomitant[Title/Abstract])) OR (Concomitant Chemoradiotherapies[Title/Abstract])) OR (Concomitant Radiochemotherapy[Title/Abstract])) OR (Concomitant Radiochemotherapies[Title/Abstract])) OR (Radiochemotherapies, Concomitant[Title/Abstract])) OR (Radiochemotherapy, Concomitant[Title/Abstract]))) OR (chemoradiotherapy followed by surgery[Title/Abstract])) OR (neoadjuvant chemoradiotherapy[Title/Abstract])) AND (((("Neoadjuvant Therapy"[Mesh]) OR (((((((((((((((((((((((((((((((((((((((((((((((Neoadjuvant Therapies[Title/Abstract]) OR (Therapy, Neoadjuvant[Title/Abstract])) OR (Neoadjuvant Treatment[Title/Abstract])) OR (Neoadjuvant Treatments[Title/Abstract])) OR (Treatment, Neoadjuvant[Title/Abstract])) OR (Neoadjuvant Radiotherapy[Title/Abstract])) OR (Neoadjuvant Radiotherapies[Title/Abstract])) OR (Radiotherapy, Neoadjuvant[Title/Abstract])) OR (Neoadjuvant Radiation Treatment[Title/Abstract])) OR (Neoadjuvant Radiation Treatments[Title/Abstract])) OR (Radiation Treatment, Neoadjuvant[Title/Abstract])) OR (Treatment, Neoadjuvant Radiation[Title/Abstract])) OR (Neoadjuvant Radiation Therapy[Title/Abstract])) OR (Neoadjuvant Radiation Therapies[Title/Abstract])) OR (Radiation Therapy, Neoadjuvant[Title/Abstract])) OR (Therapy, Neoadjuvant Radiation[Title/Abstract])) OR (Neoadjuvant Radiation[Title/Abstract])) OR (Neoadjuvant Radiations[Title/Abstract])) OR (Radiation, Neoadjuvant[Title/Abstract])) OR (Neoadjuvant Systemic Therapy[Title/Abstract])) OR (Neoadjuvant Systemic Therapies[Title/Abstract])) OR (Systemic Therapy, Neoadjuvant[Title/Abstract])) OR (Therapy, Neoadjuvant Systemic[Title/Abstract])) OR (Neoadjuvant Systemic Treatment[Title/Abstract])) OR (Neoadjuvant Systemic Treatments[Title/Abstract])) OR (Systemic Treatment, Neoadjuvant[Title/Abstract])) OR (Treatment, Neoadjuvant Systemic[Title/Abstract])) OR (Neoadjuvant Chemotherapy[Title/Abstract])) OR (Chemotherapy, Neoadjuvant[Title/Abstract])) OR (Neoadjuvant Chemotherapies[Title/Abstract])) OR (Neoadjuvant Chemotherapy Treatment[Title/Abstract])) OR (Chemotherapy Treatment, Neoadjuvant[Title/Abstract])) OR (Neoadjuvant Chemotherapy Treatments[Title/Abstract])) OR (Treatment, Neoadjuvant Chemotherapy[Title/Abstract])) OR (Neoadjuvant Chemoradiotherapy[Title/Abstract])) OR (Chemoradiotherapy, Neoadjuvant[Title/Abstract])) OR (Neoadjuvant Chemoradiotherapies[Title/Abstract])) OR (Neoadjuvant Chemoradiation Therapy[Title/Abstract])) OR (Chemoradiation Therapy, Neoadjuvant[Title/Abstract])) OR (Neoadjuvant Chemoradiation Therapies[Title/Abstract])) OR (Therapy, Neoadjuvant Chemoradiation[Title/Abstract])) OR (Neoadjuvant Chemoradiation Treatment[Title/Abstract])) OR (Chemoradiation Treatment, Neoadjuvant[Title/Abstract])) OR (Neoadjuvant Chemoradiation Treatments[Title/Abstract])) OR (Treatment, Neoadjuvant Chemoradiation[Title/Abstract])) OR (Neoadjuvant Chemoradiation[Title/Abstract])) OR (Chemoradiation, Neoadjuvant[Title/Abstract]))) AND (chemotherapy followed by surgery[Title/Abstract])) OR (preoperative chemotherapy[Title/Abstract]))) |
| Study records: |  |  |
| Data management | 11a | All statistical analyses were performed using RevMan 5.3 and Stata 15.0. The results are presented as risk ratios (ORs) with 95% confidence intervals (CIs). The heterogeneity between studies were evaluated using Cochran’s Q test and the I2 statistic. A fixed-effects model was used if heterogeneity was not significant (p>0.1, I2<50.0%); otherwise, a random-effects model was applied. The results of the meta-analysis are presented as forest plots. |
| Selection process | 11b | We identified 1326 studies in the initial database search and eliminated 417 duplicate articles and 241 reviews. We excluded 497 irrelevant research studies, 102 studies that studied NCRT combined with surgery vs surgery alone, 21 studies that studied CRT combined with surgery vs surgery alone, 5 studies that met the inclusion criteria that were in progress, 21 studies that included EAC and 4 studies that had inconsistent outcome indicators. Finally, 18 studies were included in the meta-analysis, of which seven were randomized trials and eleven were retrospective studies. |
| Data collection process | 11c | Two authors (MY and YY) independently extracted the relevant data, including authors; year of publication; country; number of cases; age; stage; NCT regimen; neoadjuvant radiotherapy dose; pCR rate; R0 resection rate; 1-, 3-, and 5-year overall survival (OS); toxicity of neoadjuvant treatment; and postoperative complications. |
| Data items | 12 | P:Diagnosis of ESCC following cytological and histopathological examination in patients.  I:Neoadjuvant Chemoradiotherapy  C:Neoadjuvant Chemotherapy  O:pCR rate; R0 resection rate; 1-, 3-, and 5-year overall survival (OS); toxicity of neoadjuvant treatment; and postoperative complications.  Acknowledgments: This work was supported by National Natural Science Foundation of China (Grant No. 81972845). |
| Outcomes and prioritization | 13 | pCR rate: pCR was an independent significant predictor for EC, and patients who achieved pCR had better progression free survival(PFS) and OS than those without pCR.  R0 resection rate:R0 resection is the most important predictor of OS in patients with EC  1-, 3-, and 5-year overall survival (OS); toxicity of neoadjuvant treatment; and postoperative complications. |
| Risk of bias in individual studies | 14 | Egger’s test, and sensitivity analysis were used to evaluate publication bias. |
| Data synthesis | 15a | pCR rate; R0 resection rate; 1-, 3-, and 5-year overall survival (OS); toxicity of neoadjuvant treatment; and postoperative complications. |
|  | 15b | The results are presented as risk ratios (ORs) with 95% confidence intervals (CIs). The heterogeneity between studies were evaluated using Cochran’s Q test and the I2 statistic. A fixed-effects model was used if heterogeneity was not significant (p>0.1, I2<50.0%); otherwise, a random-effects model was applied. |
|  | 15c | To ensure the accuracy and stability of the research, we conducted a sensitivity analysis by omitting each study to assess its effect on the overall results. |
|  | 15d | None |
| Meta-bias(es) | 16 | Egger’s test, and sensitivity analysis were used to evaluate publication bias. |
| Confidence in cumulative evidence | 17 | all the included studies were of relatively high quality |

*** It is strongly recommended that this checklist be read in conjunction with the PRISMA-P Explanation and Elaboration (cite when available) for important clarification on the items. Amendments to a review protocol should be tracked and dated. The copyright for PRISMA-P (including checklist) is held by the PRISMA-P Group and is distributed under a Creative Commons Attribution Licence 4.0.**

*From: Shamseer L, Moher D, Clarke M, Ghersi D, Liberati A, Petticrew M, Shekelle P, Stewart L, PRISMA-P Group. Preferred reporting items for systematic review and meta-analysis protocols (PRISMA-P) 2015: elaboration and explanation. BMJ. 2015 Jan 2;349(jan02 1):g7647.*
