# Supplementary material for: Survival and complications after neoadjuvant chemoradiotherapy versus neoadjuvant chemotherapy for esophageal squamous cell cancer: A meta-analysis
Source: PLoS One. 2022 Aug 5;17(8):e0271242. doi: 10.1371/journal.pone.0271242 (PMC9355212; doi:10.1371/journal.pone.0271242)
Supplement: Attachment — Submitted filename: renamed_a7691.docx [file pone.0271242.s006.docx]

**Dear Mona Academic Editor, Pathak, PhD** I have removed any funding-related text from the manuscript as per your request and have included the revised statement in the updated cover letter, thank you for your suggestion.

Thank you very much to the two reviewers for their review of our paper and for their valuable comments. I am very grateful to the reviewers for their contributions to our paper. Below is our response to reviewers comments

**Reply review one**

Thank you for your encouragement. Although there have been related studies in China, most of them are not included in all the literature. This time, we searched all Chinese and English databases since the establishment of the database. With the literature on neoadjuvant chemotherapy, I hope to draw a relatively considerable conclusion, and provide more objective evidence-based medical evidence for neoadjuvant radiotherapy and chemotherapy of esophageal squamous cell carcinoma. Thanks again for your dedication to our articles.

**Reply review two**

Thank you very much for your valuable comments to us, thank you for your efforts on our article, we will answer your questions below。

**Question one**：The authors mentioned- 'After the initial screening, the researchers read the full text to determine whether to include a study'. Apart from the inclusion/exclusion criteria the authors enlisted, which others factors determined whether to include the study or not?

**My answer:** Because we are ultimately comparing neoadjuvant chemoradiotherapy versus neoadjuvant chemotherapy for esophageal squamous cell carcinoma, there are many trials studying esophageal cancer (including esophageal adenocarcinoma and esophageal squamous cell carcinoma), so we need to read this kind of literature carefully , to see if it separately reported the efficacy of neoadjuvant chemoradiotherapy versus neoadjuvant chemotherapy for esophageal squamous cell carcinoma. If there is, we need to extract this part of the data, and this part of the data also meets our inclusion criteria.

**Question two:** Please mention a couple of lines about the 'allocation concealment' the authors mentioned.

**My answer:** Allocation hiding mainly refers to the randomness of whether the people who implement the allocation in the experiment strictly implement the results of random numbers.

**Question three:** Please consider the drawing of Kaplan-Meier curves to show the survival difference between NCRT and NCT.

**My answer:** Thank you for your suggestion, but I'm very sorry, because our research is a secondary research based on other people's published research. The survival curve we understand should be the survival curve based on one or several influencing factors in a study, but because our research is based on many published studies, currently due to our limited ability, there is no way to Survival curves were drawn based on the literature we included. We are very sorry, we feel your suggestion is good and we need to study further on your suggestion.

**Question four:** Why did the authors not correlate the Clinicopathological features and survival outcomes of patients with NCRT/NCT? This analysis could potentially add much additional value to the manuscript.

**My answer:** Thank you very much for your suggestion, we also think it is very good suggestion. As our answer to a question above, because our research is a secondary research based on the research that others have published. So what we want to do is very limited. We can only perform a statistical analysis of studies that have been reported in the included literature. Since all the literatures on the efficacy of neoadjuvant chemoradiotherapy and neoadjuvant chemotherapy for esophageal squamous cell carcinoma were included in our study, and there was no literature linking clinicopathological characteristics and survival outcomes, we had no way to complete this idea. I am very sorry, but I think your suggestion is very good, and it has great guiding significance for our future scientific research work. Thank you for your efforts in our article. Thank you again from the bottom of my heart.
